# Bumblebee pollen collection is a costly affair

**DOI:** 10.1098/rspb.2026.0973

**Published:** 2026-10-07

**Authors:** Jonathan G. Pattrick, Meredith G. Johnson, Stephen L. Buchmann

**Affiliations:** ^1^ Department of Biology, University of Oxford, Oxford, UK; ^2^ Department of Biological Sciences, North Dakota State University, Fargo, ND, USA; ^3^ Departments of Entomology, and Ecology and Evolutionary Biology, University of Arizona, Tucson, AZ, USA

**Keywords:** respirometry, floral vibration, buzz pollination, poricidal anthers, sonication, floral buzzing, efficiency, pollination energetics, metabolic rate

Pollen is an essential nutrient used by bees to rear their larvae and, minimally, for adult nutrition [1]. Across bee species, females have evolved several different methods for collecting pollen, typically using movements of their fore, mid and hind legs and mandibles. Pollen collection often involves raking movements; bees use their fore tarsi and basitarsal brushes of the forelegs to scrape pollen directly off anthers in repeated, unidirectional strokes towards the body [1–3]. Mandibles typically bite or ‘milk’ anthers at the same time, loosening pollen for the forelegs to gather. The midlegs comb pollen off the forelegs and move it posteriorly, mirroring general grooming movements (bees often hang by one leg during grooming) to pass pollen to the hind legs. The midlegs also act as stabilizers, holding onto the flower. Hind legs then pack the pollen by pressing this into dense setae (scopae) or the pollen basket of social apids (corbiculae). This general pattern varies across bee species, with some using the mid or hind legs or even the abdomen or head to rub/scrape anthers. While raking or scrabbling, female bees walk or run across inflorescences, which is energetically more expensive than resting on a flower.

A more specialized and probably the most efficient method of pollen collection is floral vibration (sonication) or buzz pollination. Here, female bees typically bite the base of a poricidal anther (e.g. *Solanum* or *Senna*) while vibrating their powerful indirect flight muscles [4]. Pollen grains are rapidly ejected as a streaming pollen cloud from the anther pores [5]. A dusting of pollen strikes the bees’ venter and is then groomed and packed. More than 500 bee species in 90 genera (out of 508 total genera) are recorded as buzz pollinators [6,7]. Therefore, around 58% of bee species belong to genera known to buzz for pollen. Some bees (e.g. *Bombus*, *Xylocopa*) also vibrate non-poricidal flowers (e.g*. Rosa*, *Argemone*) by grasping clusters of stamens to gather pollen [3,8].

During floral vibration, bumblebee (*Bombus* spp.) thoraces heat up dramatically. Female bees often buzz the same flower and/or multiple porose flowers in rapid succession. The buzzing keeps the indirect flight muscles hot and is presumably energy intensive. Yet, despite its prevalence, the true energetic costs of these floral buzzes have not previously been measured. The innovative respirometry chamber devised by Rossi *et al.* [7] provides a unique opportunity to quantify gas exchange and energy expenditures *in vivo*.

Rossi *et al.* [7] overcome a genuine technical challenge in the world of respirometry, that is measuring the energetic cost of a high-frequency and transient behaviour. Though flight metabolic rate is one of the most measured behaviours, it is difficult to measure and conventional approaches often average CO_2_ emissions over long periods, potentially obscuring bursts of intense muscular effort. Additionally, flight behaviour is highly dependent on external conditions (temperature, humidity and polarized light, to name a few), which affect CO_2_ emissions and complicate interpretation [9]. Buzz pollination presents an even greater challenge as the behaviour is often very brief, requiring precise synchronization of respirometry measurements with observations of the behaviour itself. Beyond these technical demands, there is the fundamental challenge of encouraging natural behaviours (i.e. buzzing) in a decidedly unnatural context. Despite more than a century of research on buzz pollination [10], the energetics remained unknown until now. Rossi *et al.* [7] couple flow-through respirometry, laser vibrometry and synchronized video recording to capture real-time CO_2_ production during discrete behaviours (flight take-off and floral buzzing). While substantial unknowns remain in bee foraging energetics, their approach [7] takes an important step towards filling this knowledge gap and establishes a methodological template to measure costs of other floral interactions.

Rossi *et al.* [7] report that female worker *Bombus terrestris* bumblebees that engaged in floral buzzing had a mean mass-specific metabolic rate (MSMR) of 0.293 J g^−1^ s^−1^, equivalent to 50.5 ml CO_2_ g^−1^ h^−1^. Strikingly, this puts the cost of floral buzzing almost on a par with bumblebee flight, a behaviour regarded as one of the most metabolically expensive activities proportional to body size across the animal kingdom [11–13]. Indeed, using the same bees, Rossi *et al.* [7] measured the cost of take-off events, reporting a mean MSMR only slightly, but significantly, higher of 0.317 J g^−1^ s^−1^ (54.9 ml CO_2_ g^−1^ h^−1^). While there was considerable variability in metabolic rates across measurements, this was more a reflection of high between-individual rather than within-individual variability, suggesting that metabolic costs of both take-offs and buzzing are consistent within an individual, at least within the same foraging bout. Taken together, this pattern of variability may have important implications in terms of how bees differ in their foraging efficiency when extracting pollen by vibrating flowers—a bee which can extract the same quantity of pollen for a lower energetic cost will be more efficient.

Notably, body mass is a strong predictor of metabolic rate for both behaviours, scaling with an exponent of 0.833 ± 0.218 s.e. Metabolic rate (broadly) tracks mechanical power output of flight muscles, which depends on both vibration frequency and amplitude; therefore, differences in either parameter could drive differences in cost between behaviours. Floral buzzes have a higher frequency than that of wing beats during flight, and previous research has found that this frequency difference is larger for large bees than for small bees [10]. A consequence of this could be that the scaling exponent of metabolic rate with body size is greater for buzzing than for flight, although the relationship between body size and vibration amplitude is itself inconsistent across studies [10], limiting how far this can be evaluated from frequency alone. The results from Rossi *et al.* [7] show a trend in this direction; however, they found that the scaling of body mass with metabolic rate was not significantly different during buzzing and take-off events.

Although the confidence intervals are wide, Rossi *et al.* [7] note that the shared scaling exponent is similar to previous measurements for bumblebee flight. Their estimate (0.833) is also similar to the scaling of flight metabolic rate observed across insect species and may more closely align with the metabolic-level boundaries hypothesis, which states that energetically intensive behaviours (flight or buzzing) experience different constraints than resting metabolism, and thus scale more linearly [13]. In other words, buzzing appears to be an energetically expensive behaviour across the size spectrum of bumblebees. Interestingly, metabolic rate did not scale with thorax width, suggesting that energetic demand may be driven by the complete loaded mass of the bee. However, these scaling patterns should be interpreted cautiously given the wide confidence intervals; additionally, the bees used in this study were probably minimally loaded.

Floral visits are the *raison d’être* for most bee foraging trips. The energetic cost of such visits has always been assumed to vary considerably, depending upon environmental conditions, floral morphology and whether the bee is nectar or pollen foraging. At one end of the spectrum, a bee foraging by walking around on a large open inflorescence may require minimal metabolic expenditure. At the other extreme, collecting pollen by floral buzzing is believed to be more costly. The study by Rossi *et al.* [7] not only provides a direct measure of this cost, but, to our knowledge, is the first direct measurement of the metabolic cost of any type of floral visit in bees and thus represents a benchmark for future energetics research.

Bumblebee colonies have a limited time to gather resources to ensure colony growth and reproduction; consequently, optimization of foraging energetics is crucial for success [11]. The high cost of floral buzzing identified by Rossi *et al.* [7] may therefore be a major factor shaping energy budgets, floral choice and foraging behaviour. Because buzz-pollinated plants are often nectarless, bees that expend energy on buzzing must make additional nectar foraging visits to refuel, adding both time and energetic cost to a foraging trip. This may partly explain why pollen-foraging trips last longer than nectar-foraging trips [14].

For 60 seconds of floral buzzing, Rossi *et al.* [7] estimate a refuelling cost of 0.29–1.11 µl of nectar, depending on nectar sugar composition. To assess buzzing's relative importance in overall foraging energetics, consider an average pollen foraging trip for *Bombus huntii* (41.86 minutes [14]). Assuming 5 minutes of rest (MSMR = 0.034 J g^−1^ s^−1^ [12]), with remaining time split between buzzing (MSMR = 0.293 J g^−1^ s^−1^ [7]) and flight (MSMR = 0.317 J g^−1^ s^−1^ [7]), then 60 seconds of buzzing equates to approximately 2.5% of the total trip cost. It is not until around 240 seconds of buzzing that this proportional cost reaches 10%. Thus, the duration of buzzing during a foraging trip is a key parameter for determining the energetic importance of this behaviour.

We developed a simple model to explore how buzzing time varies as a function of several key parameters. Assuming an average corbicular load of 21.2 mg [15], 20% of which is nectar (which bees mix with corbicular pollen), and that bees collect 27% of the pollen released by buzzing [16], we allow pollen mass per flower to vary from 0.5 to 6 mg and calculate the buzzing time required for a bee to obtain sufficient pollen to fill both hind leg corbiculae. This is calculated for several pollen-dispensing rates, varying from 2 to 10% of available pollen released per second of buzzing. We make the simplifying assumption that pollen release per second is always 2–10% (respectively) of the total pollen mass per flower (equivalent to a bee visiting a new flower for each additional second of buzzing). In reality, pollen release follows an exponential decay relationship [4]; however, our model represents an idealized scenario, giving an upper estimate of the amount of pollen a bee can collect per second (figure 1A).

**Figure 1.**
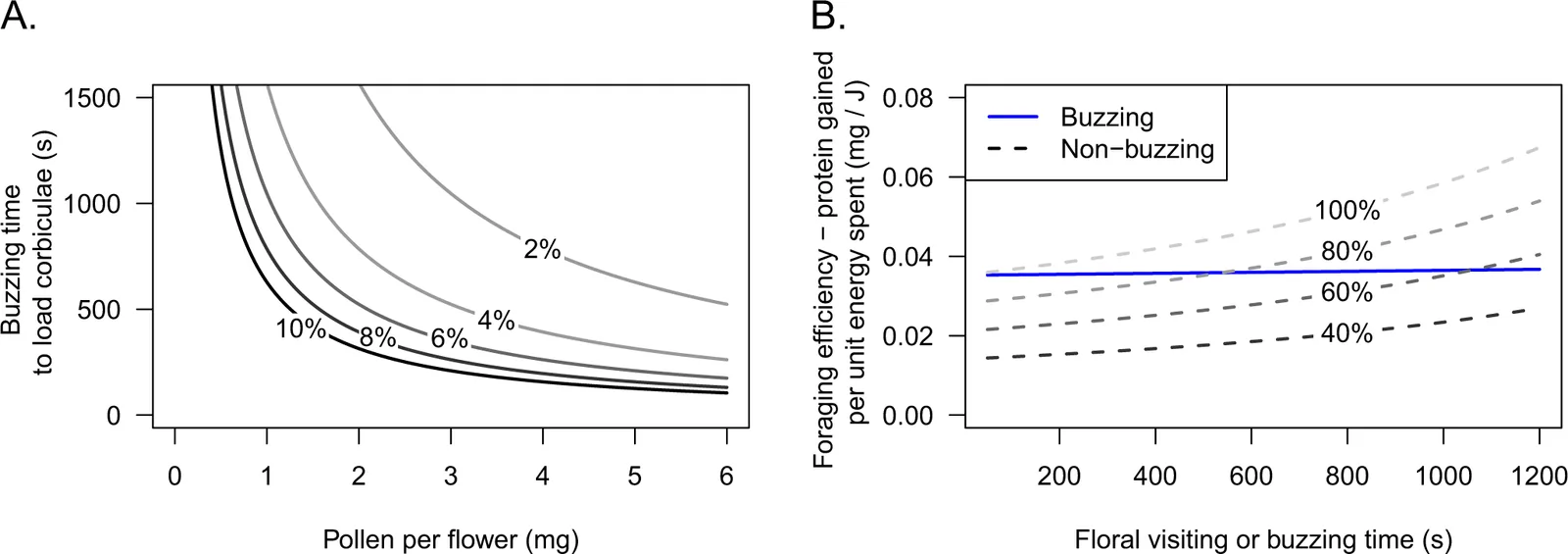
(A) Time to load corbiculae as a function of pollen mass per flower at variable dispensing efficiencies per second of buzzing. (B) Foraging efficiency for buzzing versus non-buzzing visits in terms of pollen protein gained versus energy spent. Note that in this model, the efficiency of a trip using floral buzzing (blue line) is relatively constant because of the fixed trip length and similar costs of buzzing and flight. The four dashed lines for non-buzzing visits represent foraging on pollen that is 100, 80, 60 or 40% the protein content (assumed 30% w/w) of pollen from a buzz-pollinated plant. Please see the supplementary material for the code used to generate the figure.

The model indicates a high dependence of buzzing time on both pollen mass per flower and dispensing rates. Even for a high pollen mass and 10% of pollen dispensed per second, total buzzing time is still 100 seconds, while for low pollen mass buzzing time increases exponentially (figure 1A). A buzz-pollinated *Solanum rostratum* flower can contain 164 400–693 850 pollen grains [4], which we estimate to weigh 0.8–3.3 mg and recorded dispensing rates for this species are 2–4% of remaining pollen per second, though other plant species have higher dispensing rates [4]. Bearing in mind that our model represents an optimal scenario, these predictions suggest that buzzing time may comprise a significant proportion of the energetic budget of a bumblebee foraging trip. This modelling exercise also highlights the need for more data on parameters such as pollen dispensing rates and the proportion of buzzed pollen which bees collect/store, as these will significantly affect the time and thus energetics of pollen collection through buzzing.

Given a potentially high energetic burden of floral buzzing across a foraging trip, how does buzzing compare with other types of floral visits? We explore this by extending our model to calculate foraging efficiency in terms of pollen protein gained per unit energy spent. We compare a pollen collection trip using floral buzzing to one without, if pollen collection without buzzing involves minimal cost. Using a simplified scenario with a fixed trip length and the other parameters as above, the comparative efficiency of buzzing versus non-buzzing depends once again on the duration of pollen collection. If pollen collection is only a short component of overall trip duration, then buzzing is nearly as efficient as non-buzzing (figure 1B). However, non-buzzing pollen collection becomes increasingly efficient compared to buzzing pollen collection as total floral visit duration increases. Even if floral visiting time is high, the apparent disadvantage of buzz pollination can be resolved because buzz-pollinated plant species may produce pollen in larger quantities and with a higher protein content than non-buzz-pollinated species [6]. We illustrate this by calculating the efficiency of non-buzzing pollen collection foraging trips which obtain pollen either of the same quality (100%) or of lower quality (80, 60, 40%) compared to our buzz-pollinated pollen. In this situation, foraging efficiency is increasingly pushed in favour of buzz-pollinated pollen (figure 1B). Similarly, pollen collection by buzzing may be faster than non-buzzing, especially if buzz-pollinated plants have more pollen per flower [6], and this would result in similar comparative dynamics.

The assumption that non-buzzing pollen visits are low cost may not be accurate. Bumblebee flight requires a sufficiently warm thorax. While bumblebees can allow their thorax temperature to decrease when landed on flowers, they typically ‘shiver’ to maintain temperature above the minimum required for flight. If it is cold, this thermoregulatory cost is estimated to be equivalent to flight itself [11]. Consequently, and unless ambient temperatures are warm, other types of floral visits (including those for nectar) may be just as expensive as floral buzzing.

Conversely, at high temperatures, the muscular effort required to buzz could generate substantial metabolic heat to risk overheating [10]. Heinrich's work shows that bumblebees can prevent overheating through shunting hot haemolymph from the thorax to the abdomen [11]. Buzzing in the heat is somewhat of a dual burden; there is the large mechanical cost of buzzing itself coupled with the cost of physiological temperature management.

As Rossi *et al.* [7] point out, an important future step is measuring the metabolic cost of other types of floral visits. We further suggest that it will be vital to quantify the temporal structure of foraging trips—how long bees spend in different types of flight, resting, warming up and handling flowers, as well as floral buzzing. This is necessary for understanding both the relative energetic significance of floral buzzing, and the energetic budget of bee foraging trips more generally.

Buzz pollination is widespread across bee species [10]. The metabolic costs and (probable) thermal constraints relating to this behaviour are likely to have profound ecological and evolutionary implications for this highly diverse guild of pollinators. Assuming interspecific scaling of metabolic rate during buzzing is similar to the near-isometric intraspecific scaling observed by Rossi *et al.* [7], the large-bodied (> 1 g) buzz-pollinators like *Xylocopa* spp. theoretically face immense absolute energetic demand during floral vibration in addition to flight. This problem is compounded in hot and arid climates inhabited by bees like *Centris* spp. These large bees must balance the cost of buzzing with physiological thermoregulation, potentially limiting foraging to cooler morning or evening temperatures. Smaller-bodied buzz pollinators might manage thermal load more effectively owing to a high surface area to volume ratio, but their buzzes may operate with lower absolute force and restrict which plants they may visit. It seems plausible that rigid buzzing economics could be a selective pressure that shapes morphology, behaviour and activity period of these bees.

## Supplementary Material



## Data Availability

The code used to produce figure 1 is available as supplementary material. Supplementary material is available online [17].
